# The mitochondrial negative regulator MCJ modulates the interplay between microbiota and the host during ulcerative colitis

**DOI:** 10.1038/s41598-019-57348-0

**Published:** 2020-01-17

**Authors:** Miguel Angel Pascual-Itoiz, Ainize Peña-Cearra, Itziar Martín-Ruiz, José Luis Lavín, Carolina Simó, Héctor Rodríguez, Estibaliz Atondo, Juana María Flores, Ana Carreras-González, Julen Tomás-Cortázar, Diego Barriales, Ainhoa Palacios, Virginia García-Cañas, Aize Pellón, Asier Fullaondo, Ana Mª Aransay, Rafael Prados-Rosales, Rebeca Martín, Juan Anguita, Leticia Abecia

**Affiliations:** 1 CIC bioGUNE. Bizkaia Science and Technology Park. bld 801 A, 48160 Derio, Bizkaia Spain; 2 Faculty of Science and Technology, University of the Basque Country (UPV/EHU), Leioa, Bizkaia Spain; 3 Molecular Nutrition and Metabolism, Institute of Food Science Research (CIAL, CSIC), Madrid, Spain; 4 Department of Animal Medicine and Surgery, Veterinary Faculty, Universidad Complutense de Madrid, Madrid, Spain; 5 Commensal and Probiotics-Host Interactions Laboratory, UMR1319 Micalis, INRA, Jouy-en-Josas, France; 6 CIBERehd, ISCIII, Madrid, Spain; 7 Ikerbasque, Basque Foundation for Science, Bilbao, Bizkaia Spain

**Keywords:** Acute inflammation, Ulcerative colitis

## Abstract

Recent evidences indicate that mitochondrial genes and function are decreased in active ulcerative colitis (UC) patients, in particular, the activity of Complex I of the electron transport chain is heavily compromised. MCJ is a mitochondrial inner membrane protein identified as a natural inhibitor of respiratory chain Complex I. The induction of experimental colitis in MCJ-deficient mice leads to the upregulation of *Timp3* expression resulting in the inhibition of TACE activity that likely inhibits *Tnf* and *Tnfr1* shedding from the cell membrane in the colon. MCJ-deficient mice also show higher expression of *Myd88* and *Tlr9*, proinflammatory genes and disease severity. Interestingly, the absence of MCJ resulted in distinct microbiota metabolism and composition, including a member of the gut community in UC patients, *Ruminococcus gnavus*. These changes provoked an effect on IgA levels. Gene expression analyses in UC patients showed decreased levels of *MCJ* and higher expression of *TIMP3*, suggesting a relevant role of mitochondrial genes and function among active UC. The MCJ deficiency disturbs the regulatory relationship between the host mitochondria and microbiota affecting disease severity. Our results indicate that mitochondria function may be an important factor in the pathogenesis. All together support the importance of MCJ regulation during UC.

## Introduction

The pathogenesis of inflammatory bowel disease (IBD) is multifactorial, involving the interplay between the microbiota, the intestinal luminal environment, the intestinal epithelial barrier, and both the adaptive and innate immune responses. Although the role that mitochondria dysfunction plays in IBD is not well understood, there is a significant connection between intestinal inflammation and mitochondrial function. Healthy mitochondria are important for adequate energy supply to all cellular processes, presenting patients with IBD reduced ATP levels within the intestine^1–3^. Conversely, variations in mitochondrial DNA, which result in increased concentration of intestinal ATP and augmented oxidative phosphorylation complex activity, protect mice from colitis^4^. Furthermore, mitochondria of IBD patients show morphological changes^5–7^ exhibiting the colonic epithelial cells of patients with ulcerative colitis (UC) mitochondrial alterations before other ultrastructural abnormalities are apparent in the epithelium and before the onset of mucosal inflammation^8,9^. Reduced mitochondrial function of the respiratory chain complexes II, III and IV (up to 60%) has been reported in UC patients^10^ while pediatric Crohn´s disease (CD) patients have functional defects at complexes III and IV^11^. Recently, Haberman *et al*.^12^, observed a marked suppression of mitochondrial complex I activity in active UC patients. Such mitochondrial perturbations result in increased epithelial permeability as a consequence of reactive oxygen species (ROS) production and promote transcytosis of bacteria across the epithelial layer^13^. Higher intestinal permeability has been reported in active^14^ and UC in remission^15^ further suggesting a role for mitochondrial dysfunction in the pathogenesis of IBD. In addition, results from Mottawea *et al*.^16^ indicated a regulatory relationship between mitochondria and microbiota and a disturbance of this relationship in CD patients. Methylation-controlled J protein (MCJ), encoded by the *Dnajc15* gene, is a small mitochondrial protein that negatively regulates the electron transport chain (ETC)^17,18^. Endogenous MCJ associates with complex I and acts as a natural inhibitor. MCJ deficiency results in increased complex I activity and mitochondrial membrane potential without affecting mitochondrial mass^17^. The activity of complex I is enhanced by its assembly into “respirasomes”, mitochondrial ETC supercomplexes containing complexes I, III, and IV^19^. Supercomplexes facilitate the efficient transfer of electrons minimizing electron “leak” that results in ROS production^20^. Loss of MCJ in macrophages results in increased mitochondrial respiration and elevated basal levels of ROS. The activation of the JNK/c-Jun pathway also increased, leading to the upregulation of the *Tnf* converting enzyme (*Tace*) inhibitor *Timp3*, which effectively prevents the shedding of *Tnf* (tumor necrosis factor) from the membrane. MCJ regulates the production of *Tnf* by macrophages in response to a variety of Toll-like receptor (TLR) ligands and bacteria^21^. *MCJ* was initially identified as a gene negatively regulated by methylation at CpG islands in ovarian cancer^22^, Wilms tumors^23^ and melanoma^24^. Later, IFNγ was identified as a repressor of MCJ transcription in macrophages^25^. However, the role that MCJ plays during intestinal inflammation is unknown.

In this study, we used a MCJ-deficient murine model to study the role of the mitochondrial dysfunction in experimental colitis. Loss of MCJ results in a more severe disease activity index through the regulation of cytokines. This is first reflected in gut microbiota composition and intestinal permeability and then impacted via TLR in the progression of colitis. Therefore, MCJ plays a protective function during intestinal inflammation. Understanding the role of mitochondrial modulator MCJ in the pathogenesis of UC may offer key insights into the initiation and propagation of the disease.

## Materials and Methods

### Animals and experimental design

Animal protocols were approved by the Animal Research Ethics Board of CIC bioGUNE in accordance with European and Spanish guidelines and regulations. MCJ-deficient mice on a C57BL/6 background and wild-type B6 mice (8–10 wk) were maintained under specific pathogen-free conditions with controlled temperature (21–23 °C) and 12/12-hour light/dark cycles. Mice were fed ad libitum on standard mouse chow (Global diet 2914, Harlam, Madison, USA).

Dextran sodium sulfate (DSS) (36–50 kDa; TdB Consultancy) was administered in drinking water (3%) for 6 days; then, mice were given autoclaved water for 2 days. Animal body weight, the presence of gross blood in feces, and stool consistency were individually evaluated daily by a blind technician. Each parameter was assigned a score according to the criteria proposed previously^26^ and used to calculate an average daily DAI (disease activity index).

### Transepithelial permeability assay

Mice were gavaged with 600 mg kg^−1^ body weight of FITC–dextran (4 kDa; TdB consultancy) and whole blood was collected by cardiac puncture 4 h after gavage. Blood serum was collected after centrifugation at 6000 rpm for 10 min. Serum fluorescence intensity was measured using a multi-detection microplate reader (Spectramax M2, Molecular devices) with an excitation wavelength of 485 nm and an emission wavelength of 528 nm. FITC concentration (mg ml^−1^) was calculated from a standard curve using serial dilutions of FITC–dextran.

### Myeloperoxidase activity assay

One centimeter length of the distal colon was homogenized in 50 mM phosphate buffer (pH 6.0) and 0.5% hexadecyltrimethylammonium bromide using a Precellys 24 homogenizer (Bertin Instruments). After 4 cycles of 90 seconds at 6000 rpm, 7 µl of supernatant was mixed with 200 µl of 0.02% dianisidine (Sigma-Aldrich) in 50 mM phosphate buffer, pH 6.0, and 0.0005% H_2_O_2_ (Sigma-Aldrich). Human myeloperoxidase (MPO) (Merck Millipore, cat number 475911) was used as a standard to measure samples’ activity. All activity assays were performed in triplicates on 96 well microtiter plates and analyzed with a microplate reader measuring absorbance at 450 nm (Spectramax M2, Molecular devices).

### Cell preparation

Spleens and mesenteric lymph nodes were dissected post-mortem and collected in PBS (Gibco). For splenocyte and lymph node cell preparation, organs were mashed through a 70-μm cell strainer (Falcon), and erythrocytes from spleens were lysed using ACK Lysis Buffer.

### Cell analysis by flow cytometry

Cells were stained with the following fluorochrome-conjugated antibodies: CD11b APC (Miltenyi Biotech, M1/70); CD11c PE Cy7 (Miltenyi Biotech, N418); CD103 PE (Miltenyi Biotech, REA789); F4/80 FITC (Miltenyi biotech, REA126); Fc receptors were blocked with Anti-mCD16/CD32 (BD). Only events that appeared single in forward-scatter width were analyzed. A FACSCanto II and FACSDiva software (BD) were used for flow cytometry and data were analyzed using FlowJo software (TreeStar).

### Histology and immunohistochemistry

Colon tissue was fixed in 10% formalin or Carnoy´s fixative, dehydrated, embedded in paraffin and cut into 5 μm-thick sections. For histopathology, sections were deparaffined, hydrated and stained with hematoxylin and eosin or periodic acid-Schiff (PAS) according to the standard protocol. Stained sections were analyzed by a pathologist blinded to mouse genotype and treatment. The number of goblet cells was determined on PAS stained slides and expressed as the percentage per intestinal epithelial cells. For immunofluorescent analysis, tissue sections were deparaffined, hydrated and subjected to antigen retrieval using proteinase K for 15 min at 37 °C or nothing. After blocking (3% H_2_O_2_), sections were incubated with primary antibodies for 1 h (F4/80, 1:50, Biolegend) or 2 h (Muc2, 1:100, Santa Cruz), followed by 30 min incubation with fluorescently labelled secondary antibody. Finally DAPI was added to stain nuclei. Photographs were taken with a fluorescence microscope (Axioimager.D1 Zeiss) and analyzed by two people blinded to the treatment. At least 10 visual fields were captured randomly.

Colon serial sections (5 µm) were subjected to immunohistochemistry (IHQ) with primary antibody specific for IgA, HRP conjugated (IgA; dilution 1:100). Antigen retrieval was performed incubating with proteinase K for 15 minutes at 37 °C. After incubation tissue sections were immersed in diaminobenzidine solution for 2 min and washed. Slides were counterstained in Mayer’s hematoxylin for 30 seconds. Images were captured with a Zeiss Axioimager A1 microscope and analyzed with Frida software. At least 10 visual fields were captured randomly.

### Transmission electron microscopy

Sections (2–3 mm) from distal colon were fixed in 2% glutaraldehyde in 0.12 M phosphate buffer (PB, pH 7.4), overnight at 4 °C. Then, samples were washed with 0.1 M PB (pH 7.4) and immersed in 1% OsO_4_ in 0.1 M PB overnight at 4 °C. After a washing step with distilled water, samples were stained with 0.5% uranyl acetate during 45 minutes at 4 °C. Colon slices were washed again with distilled water, dehydrated in a graded ethanol series, and embedded in propylene oxide. Afterwards, samples were incubated 45 minutes 1:1 propylene oxide and resin, and embedded in resin overnight at room temperature. Finally, samples were added to resin blocks and kept overnight at 60 °C. The 1 µm semi-thin and 60 nm ultra-thin sections were obtained on a Leica Ultracut UCT ultramicrotome. Ultra-thin sections were collected in 200-mesh copper grids and observed in a JEOL JEM 1400 Plus ETM at 100 kV.

### Determination of ROS in colon tissue sections

Samples were sectioned in a cryostat (8 µm) and incubated with MnTBAP 150 µM for 1 h at RT. The samples were then incubated with DHE (5 µM) for 30 min at 37 °C. Sections were mounted with mounting media containing DAPI. Photographs were taken with a fluorescence microscope (Axioimager.D1 Zeiss) and analyzed by two people blinded to the treatment. At least 10 visual fields were captured randomly.

### Macrophage isolation from colon

Intestinal cells were extracted using several washing steps and by enzymatic disruption mediated by collagenase type IV, a matrix degrading enzyme that breaks down intercellular matrices as described by Harusato *et al*.^27^. MACS LS magnetic column were used to select F4/80 positive cells.

### RNA extraction, cDNA synthesis, and gene expression

Colon samples were preserved in RNAlater solution. Total RNA from colon tissue was extracted using RiboZol and Nucleospin RNA kit (Macherey-Nagel) according to manufacturer’s protocol. The cDNA was synthesized by using M-MLV reverse transcriptase (Thermo). Relative gene expression to a housekeeping gene (*Rpl-19)* was determined by using RT-PCR. Amplification and detection was performed on optical grade 384-well plates in QuantStudio 6 Flex Real-Time PCR system (Thermo Fisher Scientific, Waltham, MA, USA) with PerfeCTa qPCR Tough Mix (Quantabio, Beverly, MA, USA) and specific primers at their annealing temperature (see Supplementary Table). To normalize mRNA expression, the expression of 3 housekeeping genes was measured; *Rpl-19* was ranked as the best candidate. The mRNA relative quantification was calculated using the ΔΔCt method. PCR efficiency was always between 90 and 110%.

### Human RNAseq samples analysis

Human samples were obtained from the GEO dataset related to the study of “UC Colon RNAseq subset analysis” released as part of the GSE107593 series (https://www.ncbi.nlm.nih.gov/gds), available since Apr 17, 2018. Raw read count table “GSE107593_raw_reads_BCHRNAseq” generated upon STAR alignments was downloaded and used as input for the Differential Expression (DE) analysis, carried out by DESeq. 2^28^, aimed to detect differentially expressed genes.

### TACE activity

Colon protein extracts were incubated with 10 µM of TACE FRET Substrate I (Anaspec, Fremont, CA) in black NUNC polystyrene 96-well microtiter plates (Fisher Scientific). Colon protein extracts were treated with the metalloproteinase inhibitor TAPI-2 (50 µM; Enzo Life Sciences, Farmingdale, NY) to determine non-specific TACE activity. Enzyme activity was monitored using a BioTek Synergy HT microplate fluorescence reader (BioTek, Winooski, VT) at an excitation wavelength of 355 nm and an emission wavelength of 500 nm. Results are expressed as specific activity resulting from subtracting nonspecific activity from total activity.

### TNF ELISA

The TNF levels were determined by capture ELISA using the DuoSet II kit (R&D Systems, Minneapolis, MN) according to the manufacturer’s recommendations.

### Western blot

20 µg of protein were run on SDS-PAGE, transferred to nitrocellulose membranes and tested with antibodies specific for TIMP3, TNF bound to membrane and TNFR1. Equal loading was determined using antibodies against GAPDH from Santa Cruz Biotechnology, Dallas, TX).

### DNA extraction and microbiome analysis

Colon content was collected at sacrifice. DNA was isolated from freeze-dried colon samples (40 mg) using the FavorPrep Stool DNA Isolation Mini kit (Vienna, Austria) by following manufacturer’s instructions. In addition, the lysis temperature was increased to 95 °C. Eluted DNA was treated with RNase and the DNA concentration assessed spectrophotometrically by using a NanoDrop ND-100 Spectrophotometer (NanoDrop Technologies, Wilmington, DE, USA). Purified DNA samples were stored at −20 °C until use.

Using high throughput sequencing platforms and barcoded primer sets, phylogenetic-based methods targeting the 16S rRNA gene were used to deeply characterize the microbial populations present in the colon of experimental mice. DNA extracts were used as the template for PCR-based amplification of the bacterial V4 region of the 16S rRNA gene using a barcoded pyrotagging approach at BGI (Beijing, China) using Illumina Miseq with 2 × 250 bp paired-end reads based on a standard protocol from the manufacturer.

Data processing was performed using QIIME (v.1.9.0): Quantitative Insights Into Microbial Ecology software package^29^. Sequences were clustered as operational taxonomic units (OTUs) of 97% similarity using UCLUST^30^. OTU were checked for chimeras using RDP gold database and assigned taxonomy using the Greengenes database (version 4feb2011)^31^. Richness (number of observed species) and alpha and beta diversity metrics (Chao1, Shannon index, and phylogenetic Diversity whole tree) were calculated using the QIIME pipeline. The significant fold changes of OTU’s were performed in DESeq. 2^28^. MetaCoMET was used to visualize core microbiome^32^. The significances of grouping in the PCoA plots were tested and analysis of similarity (ANOSIM) with 999 permutations. Raw sequences were deposited in the European Nucleotide Archive (ENA) under the project number PRJEB19385.

### Metabolomic analysis

Colon content was collected at sacrifice and for each 20 mg of freeze-dried tissue, 300 µL cold methanol-water (2:1, v/v) and 200 µL chloroform with internal standards were sequentially added and mixed for 10 s with a vortex. Three cycles of freezing/thawing and mechanical homogenization were performed. Samples were centrifuged for 10 min at 3000 g at 10 °C, and the methanol-water upper phase was stored at −80 °C until LC-MS analysis. LC-MS was used for broad metabolite profiling^33^. Metabolomic analyses were performed in a Waters Acquity UPLC system hyphenated to a Bruker maXis II UHR-QTOF mass spectrometer. Chromatographic separation was performed using an ACQUITY UPLC BEH C18 (2.1 mm × 50 mm, 1.7 μm) column. Mobile phase A was 0.1% formic acid in water, and the mobile phase was B 0.1% formic acid in acetonitrile. Mass detection was run in the MS scan mode from *m/z* 20 to 2000 in ESI (+). Samples were analyzed in randomized order and in duplicate. Results were processed with MZMine v.2.3^34^ and univariate and multivariate statistical analysis was performed with MetaboAnalyst 4.0^35^. Metabolite tentative identification was performed by the query of the exact mass of the detected features against online databases (HMDB, and Metlin) within a ± 10 ppm mass range.

### Statistical analysis

GraphPad software was used for statistical analysis (GraphPad Software, San Diego, CA, USA). Results were graphed as box and whisker plots with median, quartiles, and range. The significance was assessed by two ways analysis of variance (ANOVA) followed by false discovery rate (FDR) post-test correction. The significance of a result is shown by an asterisk “*” in the box to indicate DSS treatment (DSS+) versus control (DSS−). A *P* value of less than 0.05 was considered significant. Spearman´s correlation test was used to assess the relationships among gene expression and bacterial composition from the colon wall. Only correlations with a value of p < 0.05 were considered significant and only correlations with a value of p < 0.001 were represented.

## Results

### MCJ attenuates the disease activity index of DSS-induced colitis and decreases colonic tissue damage

**Figure 1:**
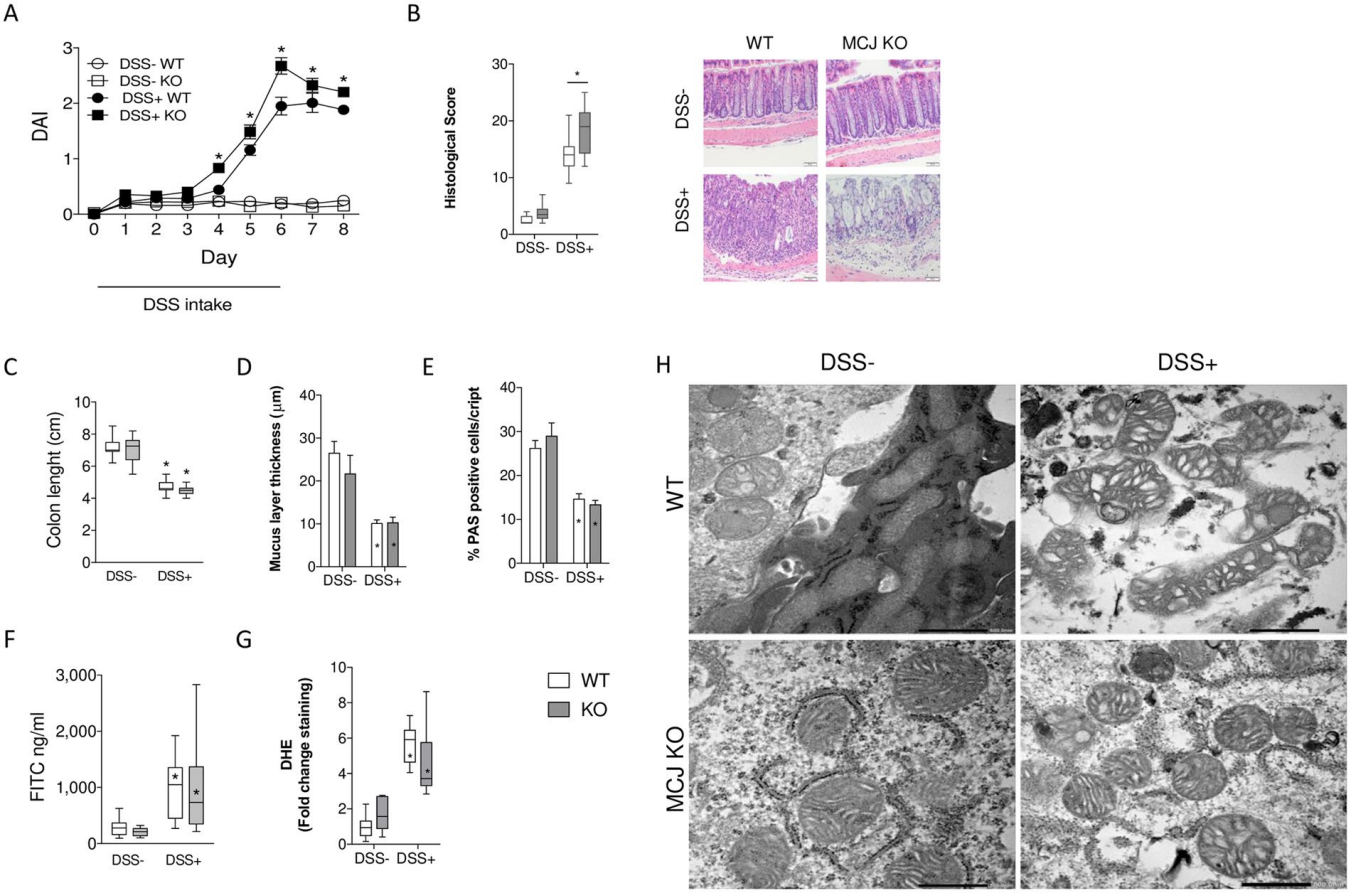
Experimental design: WT and MCJ-deficient mice received 3% DSS sterile water for 6 days, followed by 2 days of regular drinking water. (**A**) DAI values over the experimental period; data are expressed as means ± SEM (n = 20); *p < 0.05 versus DSS control group. (**B**) Histological scores. Error bars indicate SEM. Representative images. (**C**) Colon length (cm). (**D**) Mucus layer thickness (µm). (**E**) Goblet cells: % of positive cells stained with PAS (Periodic Acid-Schiff). (**F**) Permeability to the tracer FITC-dextran (ng/ml). (**G**) Reactive oxygen species (ROS) measured by dihydroethidium (DHE) staining in colon sections. (**H**) Electron microscopy showing mitochondrial morphology in DSS induced colitis groups (Scale bar: 500 nm). White: wild-type; grey: MCJ-deficient. Box and whisker plots of median, quartiles and range, n = 10 mice per group at a minimum. Statistical analysis: two-way ANOVA. “*” in boxes versus control, “*” versus WT DSS treated.

To assess the role of MCJ in the regulation of intestinal inflammatory responses, we studied a model of DSS-induced colitis. MCJ-deficient mice showed increased disease severity than WT mice, as reflected by higher disease activity index (DAI; Fig. 1A). In agreement with previous studies, the entire colon of DSS-treated mice showed histopathological changes, with loss of crypt regions. Strikingly, MCJ-deficient mice showed higher histopathology score (18.3 ± 1.29), compared to DSS-treated WT (14 ± 1.33) mice (p ≤ 0.01) (Fig. 1B).

To further assess the severity of colitis, colonic length, mucus layer thickness and the number of goblet cells were measured in all experimental groups. Both DSS-treated groups showed a marked reduction in colon length (Fig. 1C), mucus layer thickness (Figs. 1D; S1A) and number of goblet cells (Figs. 1E; S1B), and higher permeability to the paracellular tracer FITC-dextran (Figs. 1F; S1C), with no differences between WT and MCJ-deficient mice. Furthermore, analysis of ROS measured by dihydroethidium (DHE) staining in colon sections showed increased levels in both genotypes (Fig. 1G). Moreover, mitochondria shape observed by TEM showed that in MCJ-deficient mice changed from tubular to circular form with inflammation (Figs. 1H; S1D). These results suggest that MCJ function helps to temper the tissue damage upon colon injury, supporting the hypothesis that an inhibition in mitochondrial respiratory contributes to histopathological changes during colitis disease aggressiveness.

### MCJ increases colonic MPO activity and CD11c^+^CD103^+^ cells in mesenteric lymph nodes

**Figure 2:**
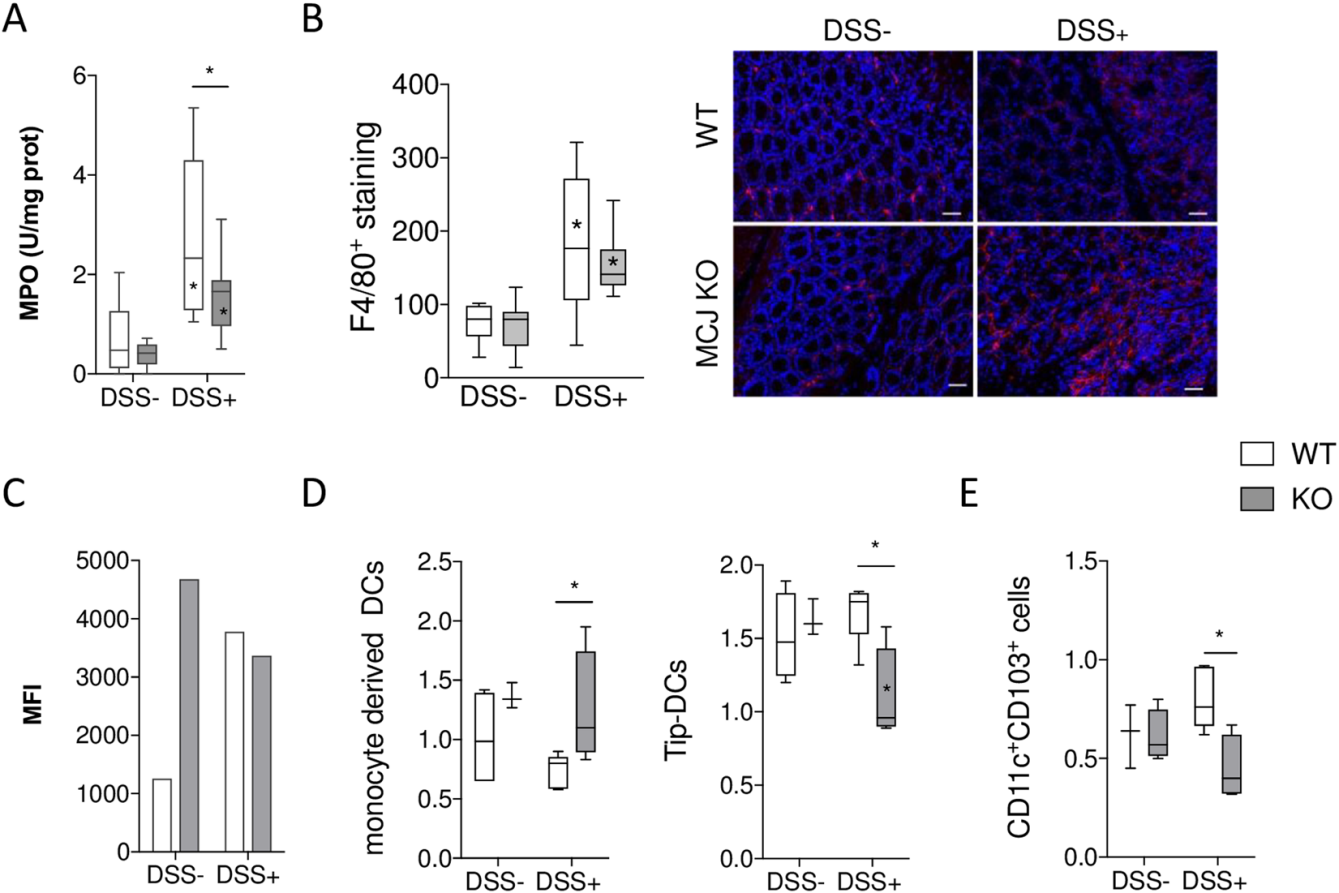
(**A**) MPO activity (U/mg protein). (**B**) Macrophage infiltration in the colon of DSS-treated mice. Representative immunofluorescence sections of colonic macrophages by F4/80 marker. Bar represents 50 µm. (**C**) The average of the mean fluorescence intensity (MFI) determined by flow cytometry showing the expression of TNF bound to membrane present in macrophages isolated from colon tissue. The values presented correspond to one experiment of two with similar results. (**D**,**E**) Flow cytometry analysis of lymphoid populations in mesenteric lymphoid nodes. White: wild-type; grey: MCJ-deficient. Box and whisker plots of median, quartiles and range, n = 7 mice per group at a minimum. Statistical analysis: two-way ANOVA. “*” in boxes versus control, “*” versus WT DSS treated.

We then analyzed the cellular composition and cell activation status in the presence or absence of MCJ. First, we evaluated the infiltration and activation of neutrophils in the local inflammatory colonic sites by measuring myeloperoxidase (MPO) levels. MPO levels were significantly increased (p ≤ 0.0001) in both WT and MCJ KO mice treated with DSS. However, DSS-treated MCJ KO mice showed (p ≤ 0.05) lower MPO activity than WT mice (Fig. 2A), suggesting a reduced infiltration of neutrophils. Macrophages infiltrated in lamina propria and submucosa were also examined by expression of the cell surface marker F4/80 in serial colon sections. The number of F4/80^+^ cells was significantly higher in the inflamed mucosa of DSS fed mice, compared to untreated mice and no obvious difference was observed between genotypes (Fig. 2B). Then, colon macrophages were isolated from tissue and TNF was determined by flow cytometry (Fig. 2C). Results showed that TNF bound to membrane levels in both DSS+ groups and MCJ deficient DSS- group were higher than WT DSS- one.

We also determined cellular populations in the spleen and mesenteric lymph nodes (MLN) by flow cytometry. We identified an increased population of CD11b^high^ and F4/80 double positive cells (monocyte-derived dendritic cells: MDDC) in spleen from mice deficient in MCJ treated with DSS (Fig. 2D). In contrast, the population of cells that were positive for CD11b and CD11c (TNF and nitric oxide producing DC, Tip-DCs) decreased. In MLN, the CD11c and CD103 (dendritic cells) positive population did not change as a result of the treatment with DSS, although we noted a small, albeit significant decrease in MCJ KO DSS-treated mice compared to DSS-treated WT mice (Fig. 2E). Overall, these results showed that innate immune populations are not dramatically changed in the absence of MCJ during colitis.

### MCJ affects gene expression in murine colonic tissue

**Figure 3:**
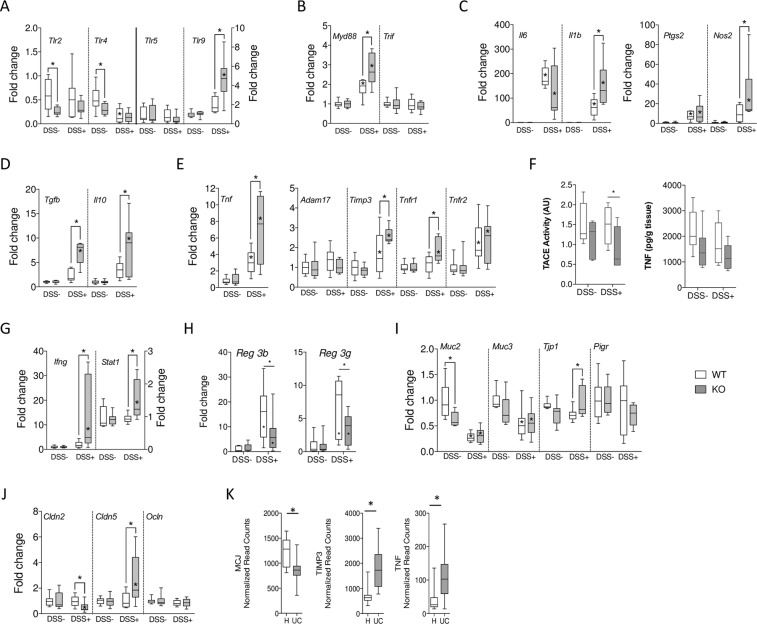
Gene expression levels in colon tissue in a DSS-induced colitis model. mRNA fold change normalized to *Rpl19* gene, and to WT (white) or MCJ KO (grey) control, respectively. (**A**) *Tlr2, Tlr4, Tlr5*, and *Tlr9*. (**B**) *Myd88* and *Trif*. (**C**) *Il6, Il1b, Ptgs2*, and *Nos2*. (**D**) *Tgfb*, and *Il10*. (**E**) *Tnf, Adam17, Timp3, Tnfr1*, and *Tnfr2*. (**F**) Tace activity and TNF concentration (**G**) *Ifng*, and *Stat1*. (**H**) *Reg3b, and Reg3g* were determined by real-time qPCR. Results are fold change normalized to *Rpl19* gene, and to WT (white) or MCJ KO (grey) control, respectively. (**I**) *Muc2, Muc3, Tjp1*, and *Pigr*. (**J**) *Cldn2, Cldn5*, and *Ocln*. Box and whisker plots of median, quartiles, and range, n = 7 mice per group at a minimum. Statistical analysis: two-way ANOVA. “*” in boxes versus control, “*” versus WT DSS treated. (**K**) Gene expression levels of MCJ, TIMP3, and TNF in a public human dataset (H = non inflamed; UC = inflamed tissue). Sample size: H = 24; UC = 24.

In order to measure the potential effect of MCJ on the inflammatory output in the colonic tissue during DSS-induced colitis, we quantified mRNA levels of several genes by quantitative RT-PCR. The expression levels of *Tlr* 2, 4, 5 and 9, the primary mucosal receptors of bacterial components, were analyzed. In untreated mice, the expression levels of *Tlr5* and *Tlr9* were not different in WT and MCJ-deficient mice, while *Tlr2* and *Tlr4* expression levels were significantly lower in the absence of MCJ (Fig. 3A). The treatment with DSS did not result in significant changes in the expression level of *Tlr2* or *Tlr5* regardless of the genotype. On the other hand, the treatment resulted in decreased expression of *Tlr4* that was not affected by the lack of MCJ. However, *Tlr9* expression levels increased in WT mice that had been treated with DSS, and the absence of MCJ resulted in a further significant increase of its expression levels (Fig. 3A). Although the expression levels of *Trif* did not change along the different conditions and genotypes, we found a significant increase in the expression levels of *Myd88* both as a consequence of the treatment with DSS and the genotype of the mice (Fig. 3B). Overall, these data suggest that the absence of MCJ implies an increased expression of key genes regulating the inflammatory output in the colon, including *Tlr9*-*MyD88*.

We then measured the cytokine and proinflammatory gene expression profile of colonic tissue. The expression levels of *Il6*, *Il1b*, *Ptgs2*, *Nos2*, *Tnf*, *Tgfb* and *Il10* were significantly increased as a result of the treatment with DSS both in WT and MCJ-deficient mice (Fig. 3C–E). The absence of MCJ caused significantly higher expression of genes such as *Nos2, Il1b*, *Tgfb*, *Il10*, and *Tnf*. In this regard, inflammation was confirmed by the ratio IL10/TNF measured by ELISA. The upregulation of *Tnf* and *Il1b* genes expression in the absence of MCJ were particularly intriguing due to its role in epithelial barrier disruption during IBD. In particular, membrane-bound *Tnf* is associated with more severe inflammatory conditions when interacting with *Tnfr1*^36^. Therefore, we investigated the expression of both *Adam17* and *Timp3*. As reported in bone marrow-derived macrophages^21^, the absence of MCJ resulted in significantly increased expression levels of *Timp3*, while the levels of *Adam17* remained invariable (Fig. 3E). However, TACE activity was significantly reduced in MCJ deficient group (Fig. 3F) indicating a functional control of the enzyme by MCJ. In accordance with slightly lower level of soluble TNF measured by ELISA (Fig. 3F). In addition, the levels of *Tnfr1* expression remained constant in WT mice when treated with DSS, as opposed to the observed increase in *Tnfr2* levels. Of note, the absence of MCJ specifically resulted in an augmented expression of *Tnfr1* (Fig. 3E). On the other hand, mRNA abundance of *Ifng* and *Stat1* was increased only in the absence of MCJ in DSS treated mice (Fig. 3G). These data support the augmented production of *Tnf* and the signaling through *Tnfr1* in the absence of MCJ. In addition, MCJ seems to be implicated in the regulation of *Ifng* and *Stat1* expression levels during inflammatory processes. Representative western blot reflecting TIMP3, TNFR1 and TNF bound to membrane protein levels in colon were presented in Fig. S2.

Studies in rodents indicate that innate recognition of bacteria or bacterial components triggers epithelial expression of secreted C-type lectins *Reg3g* and *Reg3b*. As expected, the expression level of *Reg3b* and *Reg3g* raised with DSS treatment (Fig. 3G). Interestingly the absence of MCJ in colitis-induced mice resulted in lower expression of both genes, indicating lower protective role against intestinal translocation which might be associated with the severity of the disease.

We also tested the expression of genes related to the integrity of the epithelial cell barrier, by analyzing the genes *Muc2*, *Muc3*, *Tjp1*, *Cldn2, Cldn5, Ocln* and *Pigr*. As anticipated, the expression of *Muc2* and *Muc3* were significantly repressed upon DSS treatment, but no effect was observed by the lack of MCJ (Fig. 3H,I). No differences were observed in the expression of *Tjp1* (encoding the protein *Zonula Occludens* 1). Tight junction associated genes such as *Ocln*, *Cldn2* and *Cldn5* (encoding the proteins Occludin, Claudin 2 and Claudin 5, respectively) did not show differences due to DSS treatment in WT mice (Fig. 3J). However, the MCJ-deficiency in DSS-treated mice resulted in the decreased expression of *Cldn2* and increased levels of *Cldn5* transcripts in the colon, suggesting an effect on tight junction permeability, although results should be interpreted with caution as only differences in concentration and not in localization have been determined.

In order to determine whether our findings in the murine model resemble human disease conditions, we analyzed a public dataset of UC patients (total of 48 samples) for the expression levels of MCJ. *DNAJC15* expression levels were found to be significantly lower in inflamed tissue compared to non inflamed tissue (Fig. 3K). Importantly, the expression of the genes *TIMP3* and *TNF* was increased in inflamed compared to non inflamed tissue from UC patients, strongly suggesting that MCJ expression and the subsequent regulation of downstream genes are intimately related to the inflammatory output during colonic inflammation.

### MCJ affects the composition of the host microbiome

**Figure 4:**
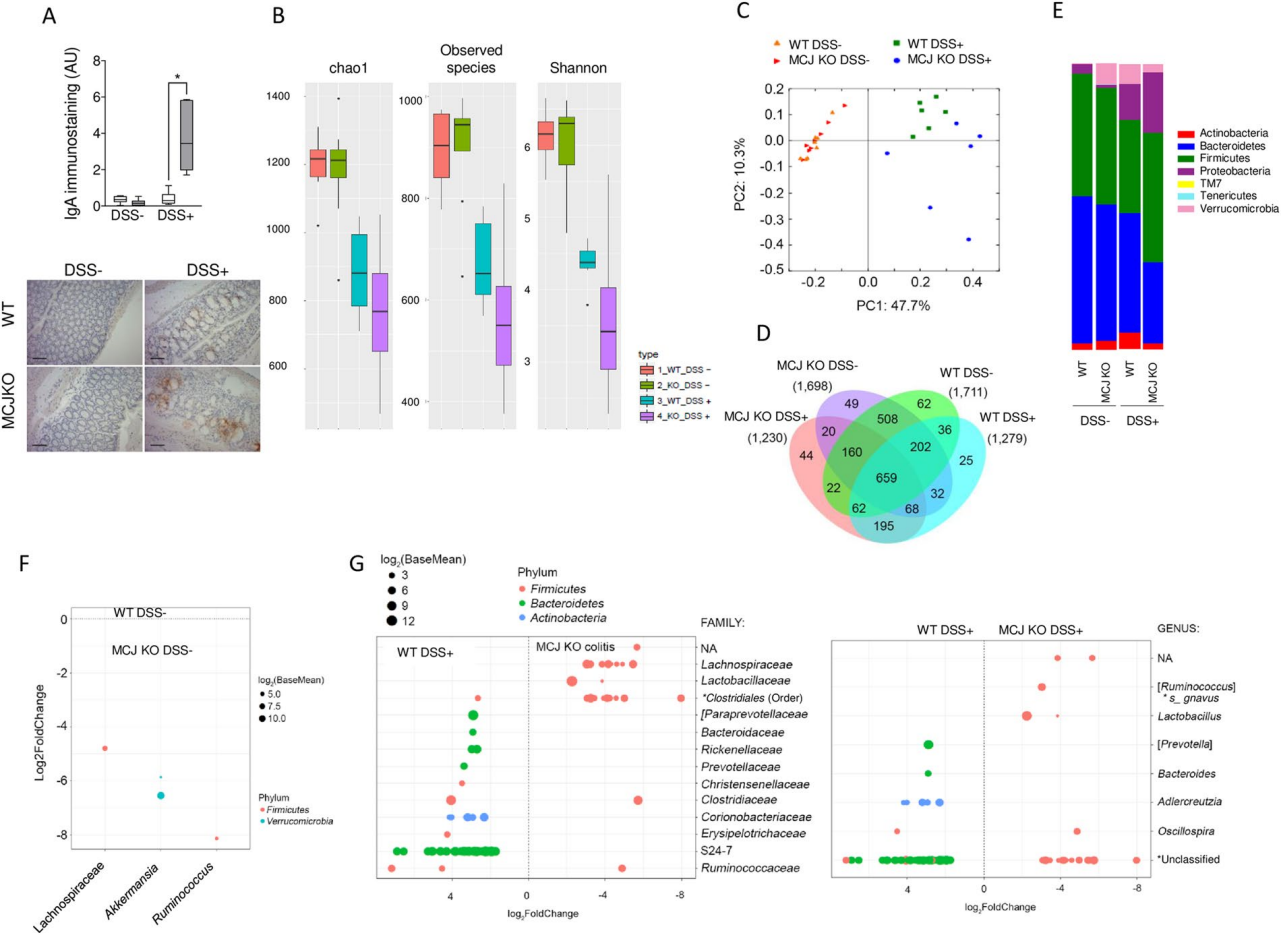
(**A**) Determination of IgA levels by immunohistochemistry in colon tissue from WT and MCJ-deficient mice during DSS-induced colitis. Error bars indicate SEM. Box and whisker plots of median, quartiles and range, n = 7 mice per group at a minimum. Statistical analysis: two-way ANOVA. “*” in boxes versus control, “*” versus WT DSS treated. (**B**) Alpha diversity measures for colon microbiomes across different experimental groups: Total observed taxonomic units, Chao1 estimates, and Shannon diversity index. (**C**) PCoA of β-diversity comparison revealed a significant separation of microbial community based on genotype in DSS experimental colitis. P = 0.01 using ANOSIM. (**D**) Venn diagram with OTUs in the colon microbiome within each experimental group. (**E**) Phylum level microbial composition. (**F**) OTUs significantly difference between WT and MCJ-deficient control groups at the genus level. (**G**) Operational taxonomic units significantly different (q < 0.05 FDR) between the colon content from WT and MCJ-deficient DSS-induced colitis groups. The left side represents OTU’s with a log2 fold positive difference for WT colon contents relative to the MCJ-deficient while the right side is the negative fold change of the WT colon relative to the MCJ-deficient contents. Each point represents a single OTU colored by phylum and grouped by taxonomic family or genus level, size of point reflects the log2 mean abundance of the sequence data.

Secretory IgA plays a role in the homeostatic maintenance of the intestinal microbiota, primarily by preventing mucosal inflammation through immune exclusion, removal of antigen-antibody complexes in the lamina propria and neutralization of inflammatory mediators. Therefore, we measured IgA levels in the colon wall by immunohistochemistry. A marked increase of IgA was found in colitis-induced MCJ-deficient mice compared to WT animals (Fig. 4A), suggesting that the absence of MCJ could shape intestinal microbiota in the inflamed colon. Therefore, we evaluated microbiota composition in animals that were induced colitis compared to healthy control mice. Illumina sequencing of the V4 region of the *16S rRNA* gene of colonic bacterial communities yielded a total of 3,161,263 paired and merged sequences after quality filtering ranging from 56,302 to 162,730 sequences per sample. A Good’s coverage average of 96.7% (range 95.3–98.2%) indicated that there was sufficient community coverage using this dataset so that the effects on the community structure of the microbiota could be assessed. Diversity indices decreased after DSS treatment presenting two indices (Observed species and Shanon) significantly lower diversity in the MCJ-deficient group (Fig. 4B). Differences were detected using principal component analysis (PCoA) of non-phylogenetic (Bray Curtis) and phylogenetic distances (weighted and unweighted Unifrac), showing changes in particular OTUs abundances. The health status of the mice provided the strongest effect on microbiota composition, followed by the presence or absence of MCJ (e.g., Bray Curtis distances; Fig. 4C). ANOSIM detected that differences between colitis-induced groups were significant (p = 0.01). Core microbiota was represented in a Venn diagram (Fig. 4D).

The comparison of taxa proportions among experimental groups identified a number of them that could be contributing to the differences observed in the microbial community. In homeostasis, the relative abundance of the phylum Verrucomicrobia was significantly higher (p = 0.008) in MCJ-deficient mice (7.1 ± 0.09%) compared to WT animals (0.05 ± 0.0001%) (n = 8 per genotype) (Fig. 4E). The examination at the genus level showed that within the phylum Verrucomicrobia only *Akkermansia muciniphila* was represented. In addition, *Ruminococcus* and a small group belonging to the Lachnospiraceae family that has yet to be assigned to a genus, were significantly increased in healthy MCJ-deficient mice (Fig. 4F). In MCJ-deficient colitis-induced mice, the analysis indicated a reduction in Bacteroidetes phylum based on the decreased levels of unclassified genus from Bacteroidetes S24–7 family, *Bacteroides* and *Prevotella*, and the increase in the presence of members of the Firmicutes phylum such as *Lactobacillus* and *Ruminococcus gnavus* (Fig. 4G). The Enterobacteriaceae family enlarged its percentage from 10 in WT to 20% in MCJ-deficient mice. The disproportionate increase in mucolytic bacteria in MCJ-deficient mice could contribute to bacterial translocation and severity of the disease.

**Figure 5:**
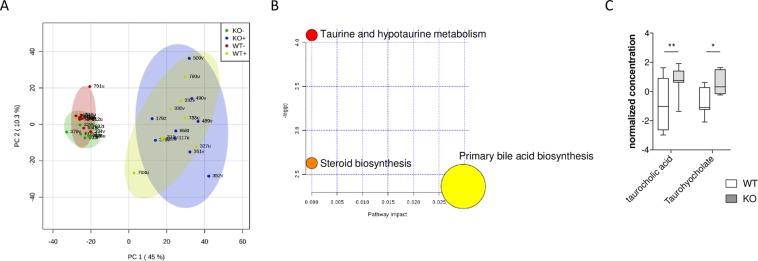
(**A**) Score plot for PCoA model with PC1 plotted against PC2 with 95% confidence ellipses around the experimental groups (KO DSS-, KO DSS+, WT DSS- and WT DSS+). (**B**) Metabolic pathway analysis. The metabolic pathways are represented as circles according to their p values from enrichment analysis (Y-axis) and pathway impact values (X-axis) using MetaboAnalyst 4.0. Darker circle colors indicate more significant changes of metabolites in the corresponding pathway (p values). The size of the circle corresponds to the pathway impact score and is correlated with the centrality of the involved metabolites. (**C**) Boxplots of the abundances of important bile acids significantly differed among the groups. The center line of each box represents the median, and the top and bottom of the box represent the 75^th^ and 25^th^ percentiles of the data, respectively. The top and bottom of the error bars represent the 95^th^ and 5^th^ percentiles of the data, respectively. n = 8 mice per group.

We then analyzed the functionality of the microbiome present in the colon of all experimental groups by a metabolomics approach using LC-MS. PCA analysis of the data showed a difference between samples from mice as a result of the treatment with DSS (Fig. 5A) with two clearly differentiated clusters: non-treated and DSS-treated mice. Supervised partial least-squares discriminate analysis (PLS-DA) was applied to further differentiate the contributions of particular metabolites to the separations between metabolite levels from WT and MCJ-deficient mice during DSS-induced colitis. Those metabolites with variable importance in the projection (VIP) > 1 were considered as potential biomarker candidates for group discrimination. The list of identified metabolites (fold changes over 1.7) was entered into the pathway analysis module from MetaboAnalyst. The results reflected significant alteration in bile acid metabolism in the digestive tract (Fig. 5B). Interestingly, MCJ-deficient mice were characterized by the higher presence of bile acids in the inflamed mucosa. More specifically, taurocholic acid and taurohyocholate (Fig. 5C) were increased in MCJ-deficient mice under inflammatory conditions. High bile acid concentration in the colon leads to aqueous stool and erratic bowel function, exacerbating diarrhea (stool consistency) which contributes to DAI. Moreover, higher levels of fecal bile acids have been identified to worsen the severity of colitis in DSS-treated mice^37^.

In summary, these data show significant shifts in the microbiota of WT and MCJ-deficient mice under inflammatory conditions, suggesting a regulatory relationship between mitochondrial function and microbiota, and a disturbance of this relationship in colitis. Moreover, our data support an association between the metabolism of bile acids, microbiota and inflammation.

### Correlation between bacterial community and gene expression in the colon under experimentally-induced colitis

**Figure 6:**
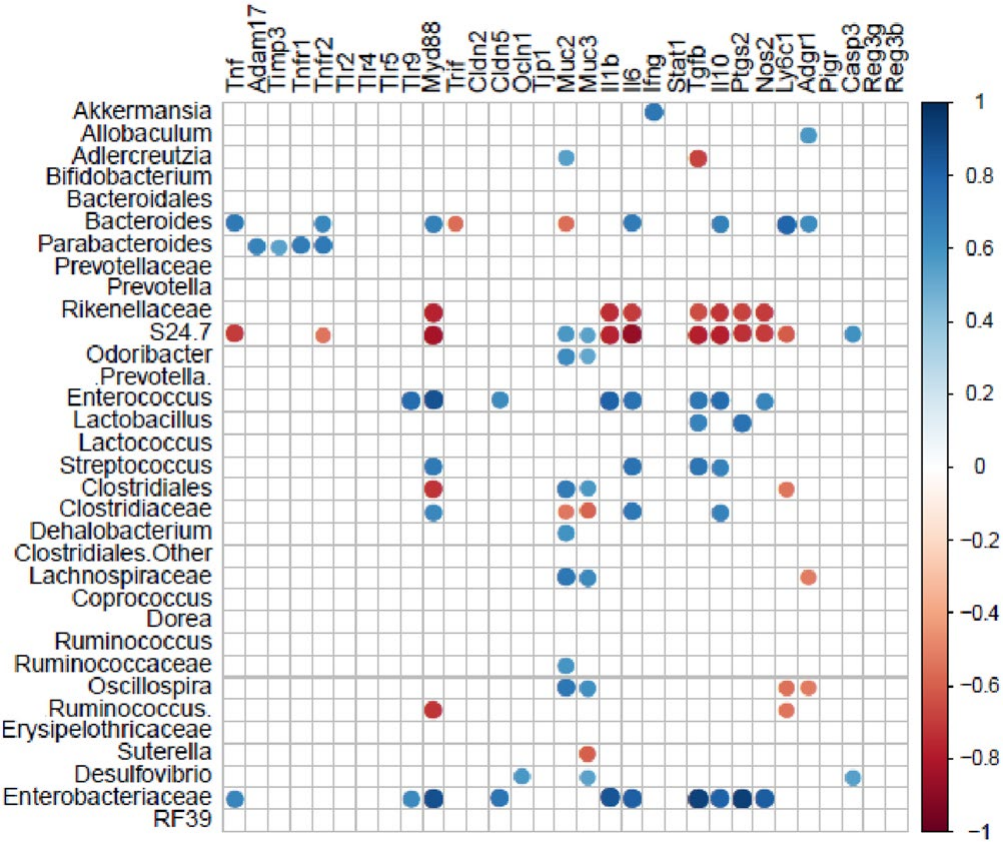
Pearson correlation of microbial community and host gene expression in the colon from WT and MCJ-deficient mice, treated or not with DSS. Pearson correlation analysis indicated that the variation in bacterial community composition and gene expression from the colon wall interaction had significant correlations (P = 0.001). The size and intensity of color for each circle represents the strength of the correlation (the larger, darker circles demonstrate a strong correlation), blue colors illustrate positive correlations and red colors illustrate negative correlation coefficient.

In order to understand whether the observed changes in microbiota composition were related to the response to colonic damage or the absence of MCJ, we performed Pearson correlation analyses. The variation in bacterial community composition and gene expression from colon wall interaction showed a significant correlation (Fig. 6). The presence of *Akkermansia* correlated (p ≤ 0.001) with *Ifnγ* expression while *Parabacteroides* correlated with the genes differentially expressed in the absence of MCJ (*Adam17, Timp3* and *Tnf* receptors). Even more important was the positive correlation observed between *Enterococcus* and the Enterobacteriaceae bacterial group with *Myd88* and *Tlr9* expression. Those bacterial groups were also correlated with the expression of cytokines such as *Il1b, Il6, Tgfb*, and *Il10*, as well as to *Ptgs2 and Nos2* expression indicating a critical role during inflammation especially in MCJ-deficient mice. On the other hand, the presence of one unclassified genus belonging to the Rikenellaceae and S24-7 families showed a negative correlation with the expression of genes associated with antigen presenting cells, pointing to a potential beneficial role of both groups of bacteria during inflammatory conditions.

## Discussion

Mitochondrial dysfunction may play an important role in the pathogenesis associated with UC. Mitochondria are the main source of ATP (adenosine triphosphate) generated through oxidative phosphorylation in the mitochondrial respiratory chain. MCJ is an endogenous negative regulator of the ETC that regulates mitochondrial function in response to altered metabolic conditions. Here, we show that the loss of MCJ results in more severe colitis, suggesting that ETC function needs to be tightly controlled to regulate the pathological consequences upon the initiation of inflammation. We also show that the absence of MCJ results in changes upon the initiation of colonic damage, which may be related to variations in intestinal microbiota-host mitochondria crosstalk and the ensuing expression levels of key genes associated with the initiation and progression of the disease.

We have identified a pathway coordinated by MCJ at gut level. MCJ absence during colitis results in the upregulation of the *Tace* inhibitor *Timp3*, which inhibits TACE activity probably affecting *Tnf* and *Tnfr1* shedding from the cell membrane. Indeed, we have reported that the loss of MCJ in macrophages inhibits *Tnf* shedding from the plasma membrane^21^. Similarly, both TNF receptors are TACE substrates^38^. In addition, transmembrane TNF induces the formation of STAT1-dependent death-inducing signaling complexes (DISC) and apoptosis through its interaction with TNFR1^39^. These data suggest that MCJ deficiency contributes to disease severity at least partly due to the regulation of membrane TNF and its ability to signal through TNFR1. More importantly was that our MCJ-deficient mouse model resembled gene expression levels of *MCJ* and *TIMP3* observed in patients with UC. However, protein levels were distinctly regulated, probably by post-translational modifications. These results indicate that the kinetics of the response is important in defining the level of disease and that two levels of functional regulation at least (transcriptional and posttranslational) are key during the development and progression of IBD.

MCJ deficiency also increased production of some cytokines related to intestinal permeability such as *Il1b*, under DSS-induced inflammatory conditions. This cytokine is a known contributor to paracellular leakage within the epithelium and the consequent breakdown of the epithelial barrier^40^. The production of proinflammatory cytokines results from the interaction of innate immune cells with bacteria translocated to the intestinal submucosa upon disruption of the epithelial barrier^41^. Upon recognition of pathogens, TLR signaling induces transcription of *Il1b*, after ROS generation and following the release of mitochondrial DNA (mtDNA)^42^. In contrast to what it was previously described in macrophages, MCJ deficiency did not affect ROS production during the inflammatory process reflecting the complex cellular interplay that takes place in colon tissue. However, our data show that not only *Tlr9* but also TLR signaling intermediate *Myd88* were augmented under disease conditions. These data are in line with those reported in the biopsy from active UC patients, in which *Tlr9* expression increased and positively correlated with the severity of intestinal inflammation as well as with inflammatory cytokine production^43,44^. The *Tlr9* cellular responses to bacterial DNA in the gut are dependent upon both the site of stimulation (apical and basolateral membrane of epithelial cells) as well as CpG sequences. Therefore, bacterial DNA from luminal bacteria that pass a leaky epithelial barrier might be an important proinflammatory agent that would contribute to the perpetuation and progression of chronic intestinal inflammation. Emerging data indicate that *Myd88* and *Tlr9* signaling of bacterial DNA is essential for induction of apoptosis and immune cell infiltration in colonic inflammation^45,46^. Moreover, *Tlr9* responds to mtDNA, which shares many similarities with immunogenic bacterial DNA due to the common evolutionary origins, contributing to inflammation^47^. Overall, these data suggest an increased susceptibility of innate immune cells to respond to invading microorganisms and the amplification of the inflammatory response. They also indicate that the absence of MCJ in mitochondria influences the production of proinflammatory cytokines such as *Il1b* through *Tlr9*/*Myd88* signaling after activation by translocated bacteria and mtDNA leaked from altered mitochondria. The activation of *Tlr9*/*Myd88* was reported to upregulate the expression of a key regulator of bile acid synthesis (FXR: Farnesoid X receptor)^48^. It is known the capacity of the gut microbiota to alter physicochemical properties of bile acids, generating high affinity ligands to host nuclear receptors such FXR^49^. In addition, previous studies^50^ suggested that taurocholate inhibits neutrophils infiltration in the inflamed colon tissue which may help to explain the decreased activity accumulation of MPO found in MCJ-deficient mice.

We found that the absence of MCJ induces changes in gut microbial community. This modulation could be related to the higher levels of bile acids described in colitis MCJ-deficient mice. Their concentration regulates microbiota in the intestine inducing negative effects on Bacteroidetes and Actinobacteria, while they exert beneficial effects on Firmicutes and Proteobacteria^51^ in agreement with our results. Therefore, the proliferation of some bile acid-tolerant microbes such as *Escherichia coli* can be facilitated by the heightened presence of these compounds^52^. In this sense, *R. gnavus* and *Lactobacillus* (phylum Firmicutes) that were augmented in colitis-induced MCJ-deficient mice, also express bile salt hydrolase indicating tolerance to bile acids^53,54^. Moreover, *R. gnavus* was proposed as an important member of the altered microbial community in IBD patients that can cope with increased oxidative stress. The increased abundance of this microorganism is often related with increased disease activity and it is also possible that contributes to the excessive immune response to microbiota^55^.

On the other hand, the defective *Muc2* mucin barrier, typical in a proinflammatory milieu, favor fast-growing bacteria that take advantage of increased sugar and oxygen concentrations induced by inflammation and tissue damage. In general, the overgrowth of Enterobacteriaceae was detected in both groups of colitis mice, with a higher increment in MCJ-deficient mice. The antimicrobial peptides and the secretory IgA balance microbial composition limiting penetration of commensal bacteria in the intestine^55^. IgA coated bacteria in IBD were associated to unclassified genus of the family S24-7 and Erysipelotrichaceae, and *Lactobacillus*^56^. All of them altered due to MCJ levels in DSS+ groups. In addition, Enterobacteriaceae abundance correlated with higher levels of IgA and lower abundance of antimicrobial peptides measured in the colon of MCJ-deficient group. According to Mirpuri *et al*.^57^ lamina propria CD11c^+^ antigen-presenting cells preferentially sample Enterobacteriaceae to produce specific IgA. All this may help to explain our results, since we detected a significant reduction in dendritic cells (CD11c^+^CD103^+^) in MLN from colitis-induced MCJ-deficient mice. This suggests that there is a higher mobilization of cells to the lamina propria to selectively uptake this group of bacteria and produce higher amounts of specific IgA in the colon of those mice.

## Conclusions

**Figure 7:**
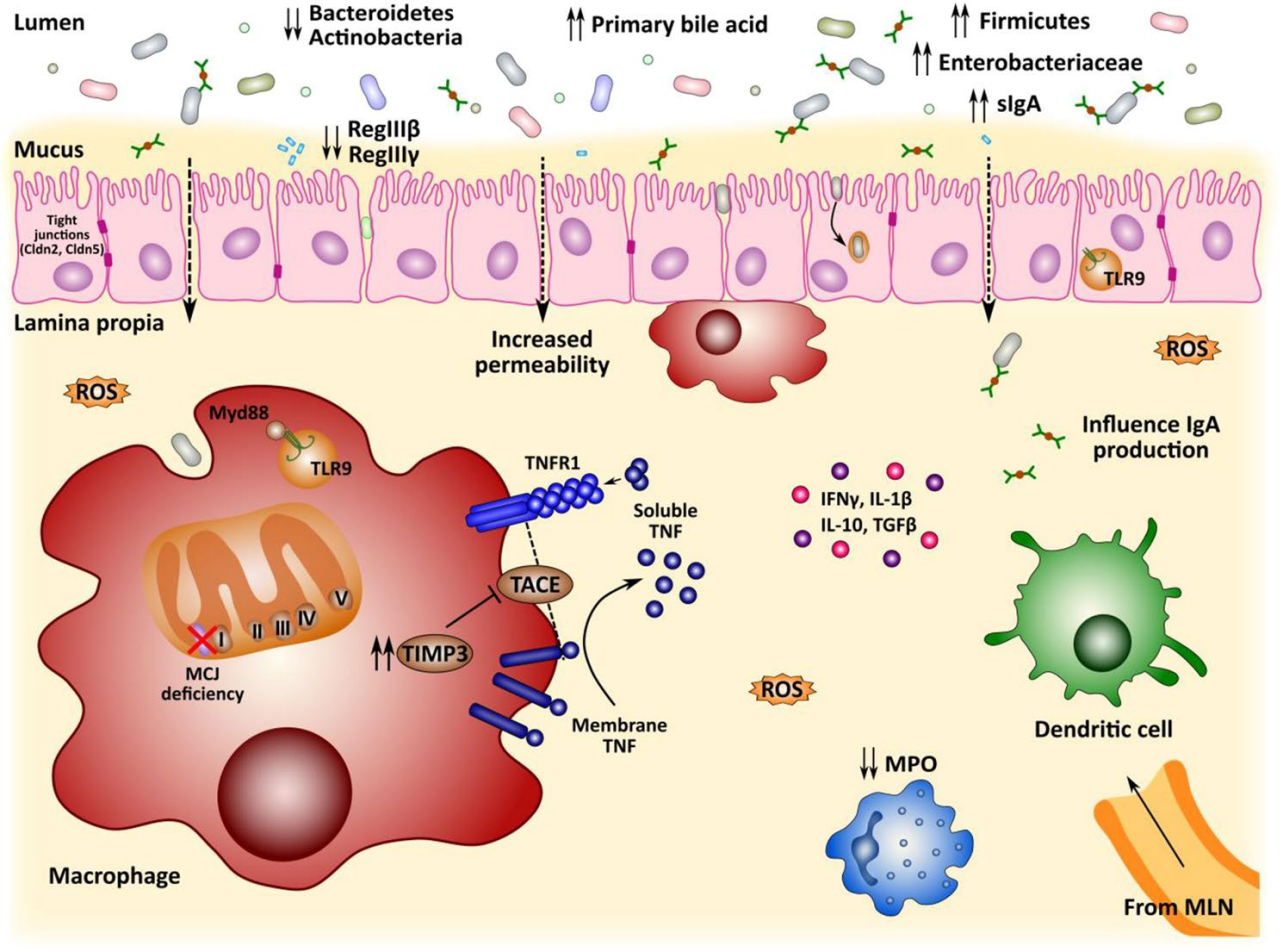
Graphical representation of potential microbiota-host interplay in DSS-induced colitis mice model deficient in MCJ.

In conclusion (Fig. 7), we observed that the loss of MCJ increased the levels of *Timp3* resulting in the inhibition of TACE activity and the shedding of membrane-bound *Tnf* and *Tnfr1*, leading to amplified *Tnfr1*-induced signaling in colon*. Tlr9*/*Myd88*-mediated signals augmented during intestinal inflammation by translocated bacteria in the absence of MCJ, with intensified proinflammatory cytokine production and a greater loss of the integrity of the colon tissue. In addition, MCJ deficiency affected bile acids levels, which modulate gut microbiota composition in colitis presenting a higher abundance of Firmicutes and Proteobacteria and lower levels of Bacteroidetes and Actinobacteria. All these factors contribute to the higher severity of the disease suggesting that dysfunctional mitochondria leads to mtDNA release potentiating inflammation in UC. Therefore, MCJ regulation is a new potential mechanism to evaluate in the disease. We propose that MCJ level links mitochondrial activity to the innate immune response and the integrity of the epithelial barrier in the gut, which indirectly regulates microbiota composition and bile acids metabolism. The differential microbiota composition together with the mtDNA leaked from mitochondrial dysfunction drives proinflammatory signaling provoking the increase in the severity of the disease. Therefore, understanding the role that MCJ plays in homeostatic and pathological conditions in the gut may open new venues for the treatment of intestinal inflammation.

### Ethics approval

Animal protocols were approved by the Animal Research Ethics Board of CIC bioGUNE (Spain; permit number CBBA-0615) and the competent authority (Diputación de Bizkaia) in accordance with European and Spanish guidelines and regulations.

## Supplementary information

Supplementary Figure 1.

Supplementary Figure 2.

Supplementary Figure 2  Full Blot Summary.

Suplementary Table 1.

## Data Availability

Raw sequences were deposited in the European Nucleotide Archive (ENA) under the project number PRJEB19385.
