# Supplementary material for: The mitochondrial negative regulator MCJ modulates the interplay between microbiota and the host during ulcerative colitis
Source: Sci Rep. 2020 Jan 17;10:572. doi: 10.1038/s41598-019-57348-0 (PMC6969106; doi:10.1038/s41598-019-57348-0)

The mitochondrial negative regulator MCJ modulates the interplay between microbiota and the host during ulcerative colitis

Miguel Angel Pascual-Itoiz[1†], Ainize Peña-Cearra[1,2†], Itziar Martín-Ruiz[1], José Luis Lavín[1], Carolina Simó[3], Héctor Rodríguez[1], Estibaliz Atondo[1], Juana María Flores[4], Ana Carreras-González[1], Julen Tomás-Cortázar[1], Diego Barriales[1], Ainhoa Palacios[1], Virginia García-Cañas[3], Aize Pellón[1], Asier Fullaondo[2], Ana Mª Aransay[1,6], Rafael Prados-Rosales[1], Rebeca Martín[5], Juan Anguita[1,7], Leticia Abecia[1*]

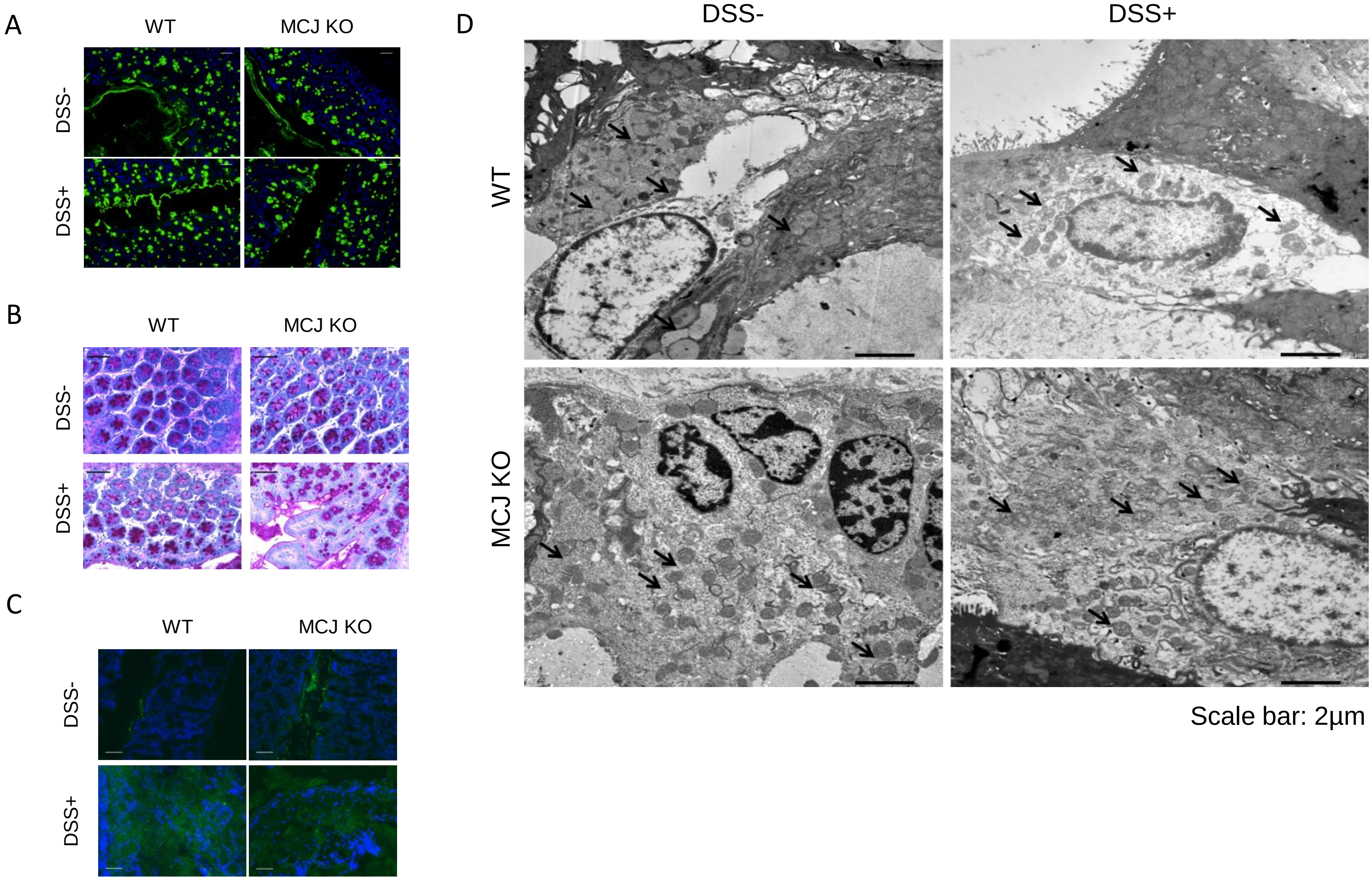

Supplement: Supplementary file 1 — Supplementary Figure 1. [file 41598_2019_57348_MOESM1_ESM.pdf]
