# Supplementary material for: The mitochondrial negative regulator MCJ modulates the interplay between microbiota and the host during ulcerative colitis
Source: Sci Rep. 2020 Jan 17;10:572. doi: 10.1038/s41598-019-57348-0 (PMC6969106; doi:10.1038/s41598-019-57348-0)
Supplement: Supplementary file 4 — Suplementary Table 1. [file 41598_2019_57348_MOESM4_ESM.pdf]

The mitochondrial negative regulator MCJ modulates the interplay between microbiota and the host during ulcerative colitis

Miguel Angel Pascual-Itoiz[1†], Ainize Peña-Cearra[1,2†], Itziar Martín-Ruiz[1], José Luis Lavín[1], Carolina Simó[3], Héctor Rodríguez[1], Estibaliz Atondo[1], Juana María Flores[4], Ana Carreras-González[1], Julen Tomás-Cortázar[1], Diego Barriales[1], Ainhoa Palacios[1], Virginia García-Cañas[3], Aize Pellón[1], Asier Fullaondo[2], Ana Mª Aransay[1,6], Rafael Prados-Rosales[1], Rebeca Martín[5], Juan Anguita[1,7], Leticia Abecia[1*]

Supplementary Table 1. Forward and reverse primer sequences for mouse *Tlr2, Tlr4, Tlr5, Tlr9, Myd88, Trif, Il-6, Il1b, Ptgs2, Nos2, Tnf, Adam17, Timp3, Tnfr1, Tnfr2, Tgfb, Il-10, Ifnγ, Stat1, Muc2, Muc3, Tjp1, Pigr, Cld2, Cldn5, Ocln1, Dnajc15, Reg3b, Reg3g,* and *Rpl19,* and qPCR conditions, reagents, instrument and quantification.

**Gene**	**Forward (F) and reverse (R) primer sequences**	**Annealing temperature (°C)**
Tlr2	F: GCAGAATCAATACAATAGAGGGAGACGC<br>R: AAGTGAAGAGTCAGGTGATGGATGTCG	60
Tlr4	F: GCAATGTCTCTGGCAGGTGTA<br>R: CAAGGGATAAGAACGCTGAGA	60
Tlr5	F: ATGGCATGTCAACTTGACTT<br>R: GATCCTAAGATTGGGCAGGT	55
Tlr9	F: CAGCTAAAGGCCCTGACCAA<br>R: GCGATCCACCGTCTTGAGAA	58
Myd88	F: CCGCCTATCGCTGTTCTTGA<br>R: GCCAGGCATCCAACAAACTG	59
Trif	F: GGACCCCACCTAGATGGCTA<br>R: CCCAAGCTAAGTCCTCTGGC	59
Il6	F: ACCACGGCCTTCCCTACTTCAC<br>R: TTCTCATTTCCACGATTTCCCAG	60
Il1b	F: ACACTCCTTAGTCCTCGGCCA<br>R: CCATCAGAGGCAAGGAGGAA	60
Ptgs2	F: GGGTTGCTGGGGGAAGAAATG<br>R: GGTGGCTGTTTTGGTAGGCTG	58
Nos2	F: GTTGAAGACTGAGACTCTGG<br>R: GACTAGGCTACTCCGTGGA	58
Tnf	F: AGCCCACGTCGTAGCAAACCAC<br>R: ATCGGCTGGCACCACTAGTTGGT	60
Adam17	F: TGGGACACAATTTTGGAGCA<br>R: CCTCCTTGGTCCTCATTTGG	60
Timp3	F: GGCCTCAATTACCGCTACCA<br>R: CTGATAGCCAGGGTACCCAAAA	60
Tnfr1	F: GCTGTTGCCCCTGGTTATCT<br>R: ATGGAGTAGACTTCGGGCCT	60
Tnfr2	F: CTCTCCAAGCCCACCGAAAT<br>R: GGACCACTGAGTTACAGCCC	60
Tgfb	F: CACTGATACGCCTGAGTG<br>R: GTGAGCGCTGAATCGAAA	60
Il10	F: TGGCCCAGAAATCAAGGAGC<br>R: CAGCAGACTCAATACACACT	58
Ifng	F: TGGTGACATGAAAATCCTGCAGAG<br>R: GCTTATGTTGTTGCTGATGGCCTG	59

Stat1	F: TCTGAATATTTCCCTCCTGGG	60
	R: CGGAAAAGCAAGCGTAATCT	
Muc2	F: GATAGGTGGCAGACAGGAGA	58
	R: GCTGACGAGTGGTTGGTGAATG	
Muc3	F: CGTGGTCAACTGCGAGAATGG	58
	R: CGGCTCTATCTCTACGCTCTC	
Tjp1	F: GTTGGTACGGTGCCCTGAAAGA	58
	R: GCTGACAGGTAGGACAGACGAT	
Pigr	F: CGAAGCTACAAGGGAGCCAA	60
	R: ACGGATAGTGGCAGGAAACG	
Cldn2	F: AGGACTTCCTGCTGACATCCAG	58
	R: AATCCTGGCAGAACACGGTGCA	
Cldn5	F: TGACTGCCTTCCTGGACCACAA	60
	R: CATACACCTTGCACTGCATGTGC	
Ocln1	F: TGGCAAGCGATCATACCCAGAG	58
	R: CTGCCTGAAGTCATCCACACTC	
Dnajc15	F: ACGCCGACATCGACCACACAG	58
	R: AATCTTCCTTGCTGTTGCCGTG	
Reg3b	F: TACTGCCTTAGACCGTGCTTTCTG	60
	R: GACATAGGGCAACTTCACCTCACA	
Reg3g	F: TTCCTGTCCTCCATGATCAAAA	60
	R: CATCCACCTCTGTTGGGTTCA	
Caspase-3	F: ATGGAGAACAACAAAACCTCAGT	60
	R: TTGCTCCCATGTATGGTCTTTAC	
Rpl19	F: GACCAAGGAAGCACGAAAGC	60
	R: CAGGCCGCTATGTACAGACA	
**Conditions**	Hold cycle 95°C for 2.30', and then 40x (95°C for 15", 60°C for 1´)	
**Reagents**	PerfeCTa SYBR® Green SuperMix (Quantabio)	
**Instrument**	QuantStudio 6 Flex Real-Time PCR System	
**Expression**	Normalized to *Rpl19* housekeeping gene using the Pfaffl equation and expressed relative to the mean of a relevant control group	
